# 1352. Exploring spatial and temporal trends in the incidence of Lyme borreliosis in Finland using surveillance data, 2015 - 2020

**DOI:** 10.1093/ofid/ofac492.1181

**Published:** 2022-12-15

**Authors:** Jozica Skufca, Nick DeSmedt, Andreas Pilz, Andrew Vyse, Elizabeth Begier, Margarita Riera, Maxim Blum, Bradford Gessner, James Stark

**Affiliations:** P95, Leuven, Vlaams-Brabant, Belgium; P95, Leuven, Vlaams-Brabant, Belgium; Pfizer Corporation Austria, Vienna, Wien, Austria; Pfizer UK, Tadworth, England, United Kingdom; Pfizer Vaccines, Dublin, Dublin, Ireland; P95, Leuven, Vlaams-Brabant, Belgium; P95, Leuven, Vlaams-Brabant, Belgium; Pfizer US, New York, New York; Pfizer US, New York, New York

## Abstract

**Background:**

Lyme borreliosis (LB) is a tick-borne zoonotic disease endemic in many European countries, including Finland. We describe the incidence, time trends and geographical distribution of LB in Finland for the period 2015–2020. The data generated can help inform public health policy, including prevention strategies.

**Methods:**

We retrieved online available LB cases and incidence from two Finnish national databases. Microbiologically confirmed disseminated LB cases were identified from The National Infectious Disease Register (NIDR) and clinically diagnosed LB cases, from the National Register of Primary Health Care Visits (Avohilmo), with the total LB cases equal to the sum from these two sources. Both registers contain data from the entire country and by hospital districts (HDs) and municipalities. Total LB incidence was calculated as the sum of microbiologically confirmed and clinically diagnosed LB cases divided by the population size.

**Results:**

A total of 33,185 LB cases were reported over the 2015-2020 period, of which 12,590 (38%) were microbiologically confirmed and 20,595 (62%), clinically diagnosed. The average annual national incidence for total, microbiologically confirmed and clinically diagnosed LB were, respectively, 99.6, 38.1, and 61.4 per 100,000 population. The overall LB incidence was highest in the south to southwestern coastal areas by the Baltic Sea and in eastern areas, with average annual incidences of 109.0 to 207.3/100,000. The Ahvenanmaa Island was a hyperendemic region with an average annual incidence of 2,473.9/100,000 and the only HD to report more microbiologically confirmed cases (69%) as compared to clinically diagnosed cases. The highest incidence rates were among persons aged over 60 years, peaking at age 70–74 years. Below 40 years, the highest incidence rates were observed among 5-9-year-olds. Most cases were reported between May and October, with a peak in July and August.

Average annual incidence (per 100,000 residents) by the Finnish hospital districts (HD) from 2015-2020. A) Clinically diagnosed LB; B) Microbiologically confirmed LB; C) Both combined, clinically diagnosed, and microbiologically confirmed LB.

**Figure:**
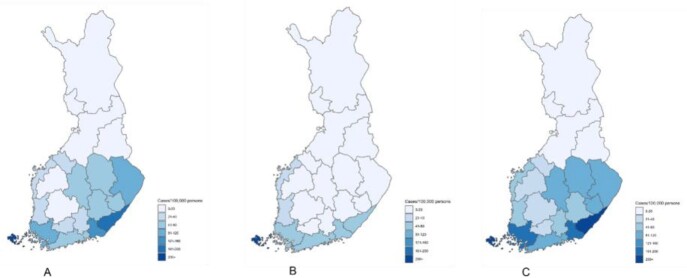

**Conclusion:**

The incidence of LB in Finland is among the highest in Europe but varied substantially by HD and many regions reported incidence much higher than the national average. These regions also corresponded to high population density areas, suggesting preventive measures such as vaccines may be an efficient use of resources.

**Disclosures:**

**Jozica Skufca, Epidemiologist**, p95: Paid by Pfizer to perform the study **Nick DeSmedt, Masters in Computer Engineering**, P95: P95 was paid by Pfizer to perform the study **Andreas Pilz, PhD**, Pfizer: Employee|Pfizer: Stocks/Bonds|Pfizer: Stocks/Bonds **Andrew Vyse, Ph.D.**, Pfizer: Stocks/Bonds **Elizabeth Begier, M.D., M.P.H.**, Pfizer: Employee|Pfizer: Stocks/Bonds **Margarita Riera, MD, MPH**, P95: Paid by Pfizer to perform the study **Maxim Blum, Ph.D.**, P95: Paid by Pfizer to perform the study **Bradford Gessner, MD, MPH**, Pfizer: Stocks/Bonds **James Stark, Ph.D.**, Pfizer: Stocks/Bonds.

